# Supplementary material for: The gene encoding ornithine decarboxylase for putrescine biosynthesis is essential for the viability of Fusobacterium nucleatum
Source: J Bacteriol. 2025 Dec 5;208(1):e00387-25. doi: 10.1128/jb.00387-25 (PMC12826042; doi:10.1128/jb.00387-25)
Supplement: Tables S1 and S2 — Strains, plasmids, and primers. [file jb.00387-25-s0001.docx]

**The Gene Encoding Ornithine Decarboxylase for Putrescine Biosynthesis Is Essential for the Viability of *Fusobacterium nucleatum***

**Shiqi Xu, Bibek G C^†^, Alex Phan & Chenggang Wu^†^**

*Department of Microbiology & Molecular Genetics, the University of Texas Health Science Center, Houston, TX, USA*

**^†^** To whom correspondence should be addressed. Tel. (+1) 713 500 5437; E-mail: [bibek.gc@uth.tmc.edu](mailto:bibek.gc@uth.tmc.edu) and [chenggang.wu@uth.tmc.edu](mailto:chenggang.wu@uth.tmc.edu)

Running Title: The *oda* gene is essential in *F. nucleatum*

Keywords: *Fusobacterium nucleatum*, ornithine decarboxylase, putrescine, polyamine, essential genes

**Supporting Tables**

**Table S1:** Bacterial strains and plasmids used in this study.

| **Strain & Plasmid** | **Description** | | **Reference** |
| --- | --- | --- | --- |
| **Strains** |  | |  |
| *Fn* subsp. *nucleatum* 23726 | Parental strain (wild-type strain) | | From ATCC |
| *Fn* subsp. *nucleatum* CTI-2 | Parental strain | |  |
| *Fn* subsp. *vincentii* 51190 | Parental strain | | From ATCC |
| *Fn* subsp. *polymorphum 10953* | Parental strain | | From ATCC |
| *Fn* subsp. *nucleatum* SX01 | *oda*::*luc*; an isogenic derivative of 23726 | | This study |
| *Fn* subsp. *nucleatum* SX02 | ∆*oda* | | This study |
| *Fn* subsp. *nucleatum* SX02a | SX02 with p*oda*(1-783aa) | | This study |
| *Fn* subsp. *nucleatum* SX02b | SX02 with p*odc* (1-502aa) | | This study |
| *Fn* subsp. *nucleatum* SX02c | SX02 with p*arg* | | This study |
| *Fn* subsp. *nucleatum* SX03 | ∆*oda* pCWU6a-*oda* | | This study |
| *Fn* subsp. *nucleatum ZP01* | ∆*tnaA* | | (1) |
| *E. coli* SZU604 | Expression of 3-methyltransferase from ATCC25586 | | (2) |
| *E. coli* DH5a | Cloning host | |  |
| **Plasmids** |  | |  |
| pCWU6 | *E. coli*/*Fusobacterium* shuttle vector, chloramphenicol /thiamphenicol resistance; cm^R^/thia^R^ | | (3) |
| pCM-galK | Suicide plasmid with GalK as a counterselection marker | | (4) |
| pCM-galK-*oda::luc* | Luciferase gene *luc* was transcriptionally fused to 3’ end of *oda* | | This study |
| pBCG02 | Deletion plasmid with HicA as the selection marker | | (1) |
| pBCG02-∆*oda* | A pBCG02-based plasmid for deletion of *oda* | | This study |
| pG106 | Shuttle for Bacteroides with *ermF/ermAM* | | (5) |
| pCWU6a | The *catP* gene in pCWU6 was replaced by the ermF/ermAF cassette from pG106 | | This study |
| pCWU6a-*oda* | Expression of *oda* gene with its original promoter in pCWU6a | | This study |
| pZP4C | The CRISPRi plasmid that targets *ftsX* | | (6) |
| pZP4C-mCherry | pZP4C with *mcherry* for generating a new CRISPRi plasmid | | (6) |
| pZP4C(*oda*) | The CRISPRi plasmid that targets *oda* | | This study |
| pBCG11 | A small shuttle plasmid for *E. coli*/*Fusobacterium* | | (7) |
| po*da* | pBCG11 expressing the whole length of *oda* (1-783aa) | | This study |
| p*odc* | pBCG11 expressing 1-502aa of ODA | | This study |
| p*arg* | *pBCG11 expressing 1-20 and 490-783aa of ODA* | | This study |
|  |  |  | |
|  |  |  | |
|  |  |  | |
|  |  |  | |

**Table S2:** Primers and DNA oligos used in this study

| **Primer** | **Sequence^1^** | | **Use for** |
| --- | --- | --- | --- |
| 2pCM-galK-F | CTAGGCTAAATATGCTTAAAAGAGatatt | | pCM-*galK-oda::luc* |
| 2pCM-galK-R | GGGCTTGGTTGTGTTGCTTTTCGGGGCTT | | pCM-*galK-oda::luc* |
| oda-F | ATTTAATATCTCTTTTAAGCATATTTAGCCTAGGATGGTGGAAAACTTAAAGGTATG | | pCM-*galK-oda::luc* |
| oda-R | TGTTACCTCCTTAGCTTAATAATCTGGGTTCATCATATATTGTACTATAC | | pCM-*galK-oda::luc* |
| luc-F | AACCCAGATTATTAAGCTAAGGAGGTAACAACAAGATG | | pCM-*galK-oda::luc* |
| luc-R | ATTTAAGCCCCGAAAAGCAACACAACCAAGCCCCTACAGTATTTAAAGATACCCCAAG | | pCM-*galK-oda::luc* |
| BCG02Mch-F | AAACGACATTGCAACACCATTGCTAAGTG | | pBCG02--∆*oda* |
| BCG02Mch-R | GGACGAGCTGTACAAGTAGTAGGG | | pBCG02--∆*oda* |
| oda-upF | ACTTAGCAATGGTGTTGCAATGTCGTTTGGTTGTGTTAATATGAATTTAATAT | | pBCG02--∆*oda* |
| oda-upR | GCACCAAAGATAACAATATTTCTTGGATTTATAATAAGAACAGCTG | | pBCG02--∆*oda* |
| oda-dnF | GCAGCTGTTCTTATTATAAATCCAAGAAATATTGTTATCTTTGGTGCGAG | | pBCG02--∆*oda* |
| oda-dnR | GATCCCTACTACTTGTACAGCTCGTCC ATATAAAGAAAGAATGAGAGATT | | pBCG02-∆*oda* |
| det-odaF | TGATACTTCATATAATAATTGAAGG | |  |
| det-odaR | GATGGCTATTTATTAGATACTATTG | |  |
| P1(oda) | GTAGTTAATTTAAAATGGCCAGATATGATGGAGAAGTTGTAGTTTTAGAGCTAGAAATAG | | pZP4C-cad |
| P2 | TAAAGTTTTATTAAAACTTATAGGATCCGCGGCCGCTA | | pZP4C-cad |
| 6a-ermF | TCCTTTATTTGTGTGATATCTAATAATTTATCTACATTCCCTTTAGTA | | pCWU6a |
| 6a-ermR | AGCATGACCGTTAAAGTGGATATCATGGTGCACTCTCAGTACAATCTGCTCTG | | pCWU6a |
| pCWU6a-F | GGACGATGGCACATAACAAACGCCGTAAACA | | pCWu6a-*oda* |
| pCWU6a-R | GTAGTAGGGATCCTCTCGAGCAGATCTC | | pCWu6a-*oda* |
| oda-G-F | TGTTTACGGCGTTTGTTATGTGCCATCGTCCTCTAAATTAGACCAAAATAAGA | | pCWu6a-*oda* |
| oda-G-R | GAGATCTGCTCGAGAGGATCCCTACTACAAGAAAATTATAAAGGAACTGTTAT | | pCWu6a-*oda* |
| pBCG11-F | TTAAAAAGACTTTAGAAGAATTAAAAGCG | |  |
| pBCG11-R | TAGCTGATGGTGCTAAAAAAATAAACTAGG | |  |
| com-odaF | TATACCTAGTTTATTTTTTTAGCACCATCAGCTAAGAGAATAATGTTCCCAGAAAACTATG | | pBCG11-*oda* |
| com-odaR | AATCGCTTTTAATTCTTCTAAAGTCTTTTTAAAAGAAAATTATAAAGGAACTGTTAT | | pBCG11-*oda* |
| oda1-502-F | ttaATTTTTCATCTTTTCAGTGTATAAG | | pBCG11-*odc* |
| oda1-502-R | TAAAAAGAAAAAGGAATTGTTATAAAATT | | pBCG11-*odc* |
| oda(arg)-F | cacatattcatcttttaatactgtaaataa tg | | pBCG11-*arg* |
| oda(arg)-R | GAAGACGCTATTTACTTATACACTG | | pBCG11-*arg* |
|  |  | |  |
|  | |  |  |
| **RT-PCR** | |  |  |
| RT-07450-F | | ACATACACAGTTAATAATGTTCCTTCAT |  |
| RT-07450-R | | GAATAGGGCTGATGTCATATAAGGT |  |
| RT-oda-F | | TCTGGTGCTATACAAGCAATGA |  |
| RT-oda-R | | AATTCCTGCTGAAACTGATTTATGA |  |
| RT-fruR-F | | ACCTTGGCTATTGTTGGAGTAA |  |
| RT-fruR-R | | GCCTCTTCTATATCATGGGTTGA |  |
| RT-ldcA-F | | CAAAGGAAAGAGCAGAGGAAATG |  |
| RT-ldcA-R | | CCTCCTATCACAGGCATCATAATA |  |
| RT-gyrB-F | | CACCATTTGGTGTGGGAAATAG |  |
| RT-gyrB-R | | TTCCTCTTCCATTGTCCATAACT |  |
| RT-02945F | | ACCTATGACAGAGGGCTATATCT |  |
| RT-02945R | | GTTCCTCCTCCAACTCCATTT |  |
| RT-pelG-F | | TGGCTGGTATAGGCTTTGAATTA |  |
| RT-pelG-R | | CCATGGGCCAACACTTACA |  |
| RT-gfrB-F | | TTCATGGGCAGGTGGTTT |  |
| RT-gfrB-R | | TCATCTGTTGCTACACTGTCAT |  |
| RT-eutC-F | | GTTGGACTTGCAGATGATGTTT |  |
| RT-eutC-R | | GCACTCATACTTTCAGCTGTTG |  |

**Fig.S1**: BLAST alignment of ODC proteins from *F. nucleatum* strains ATCC 25586 and ATCC 23726 demonstrating identical amino acid sequences.


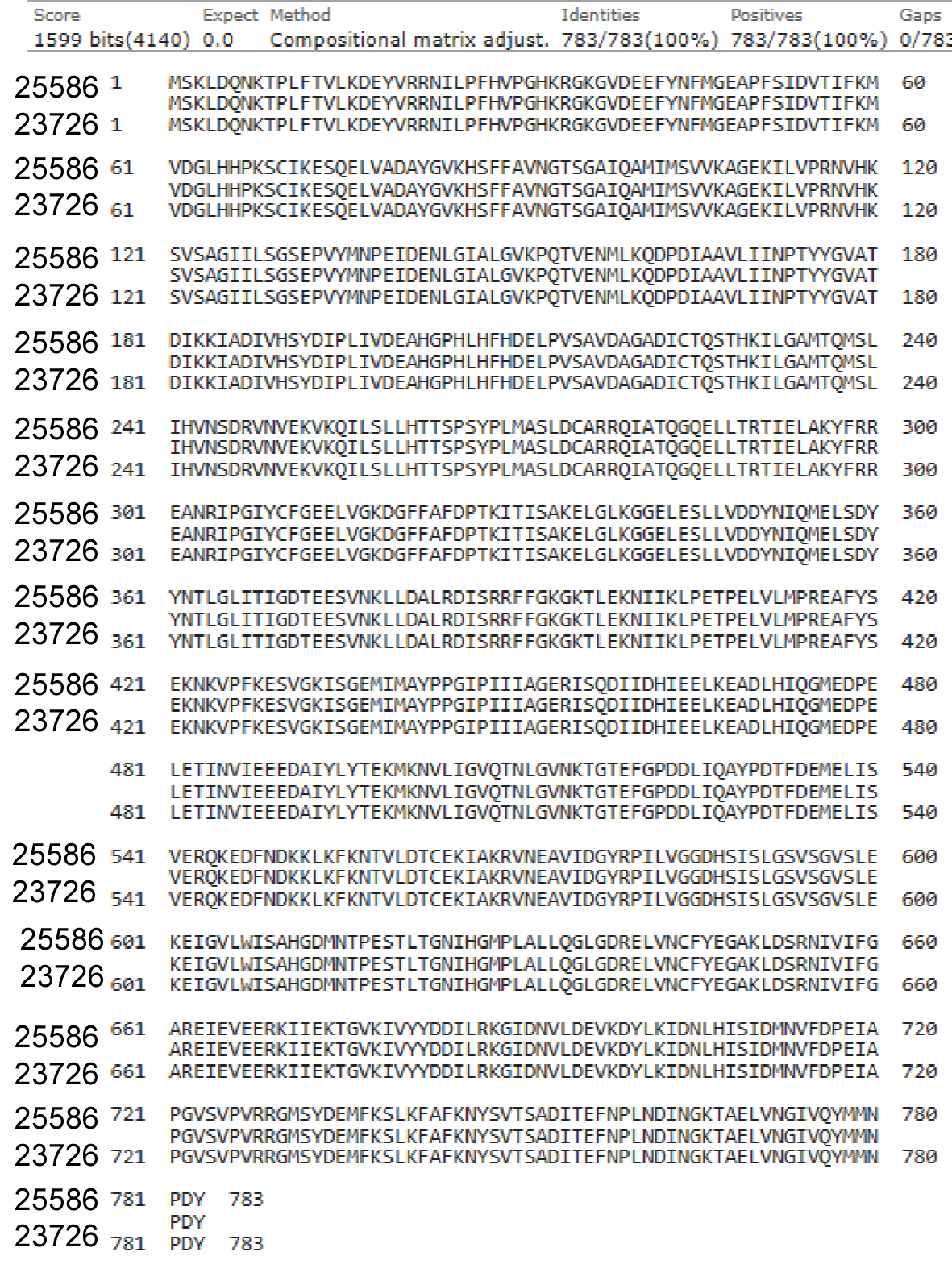


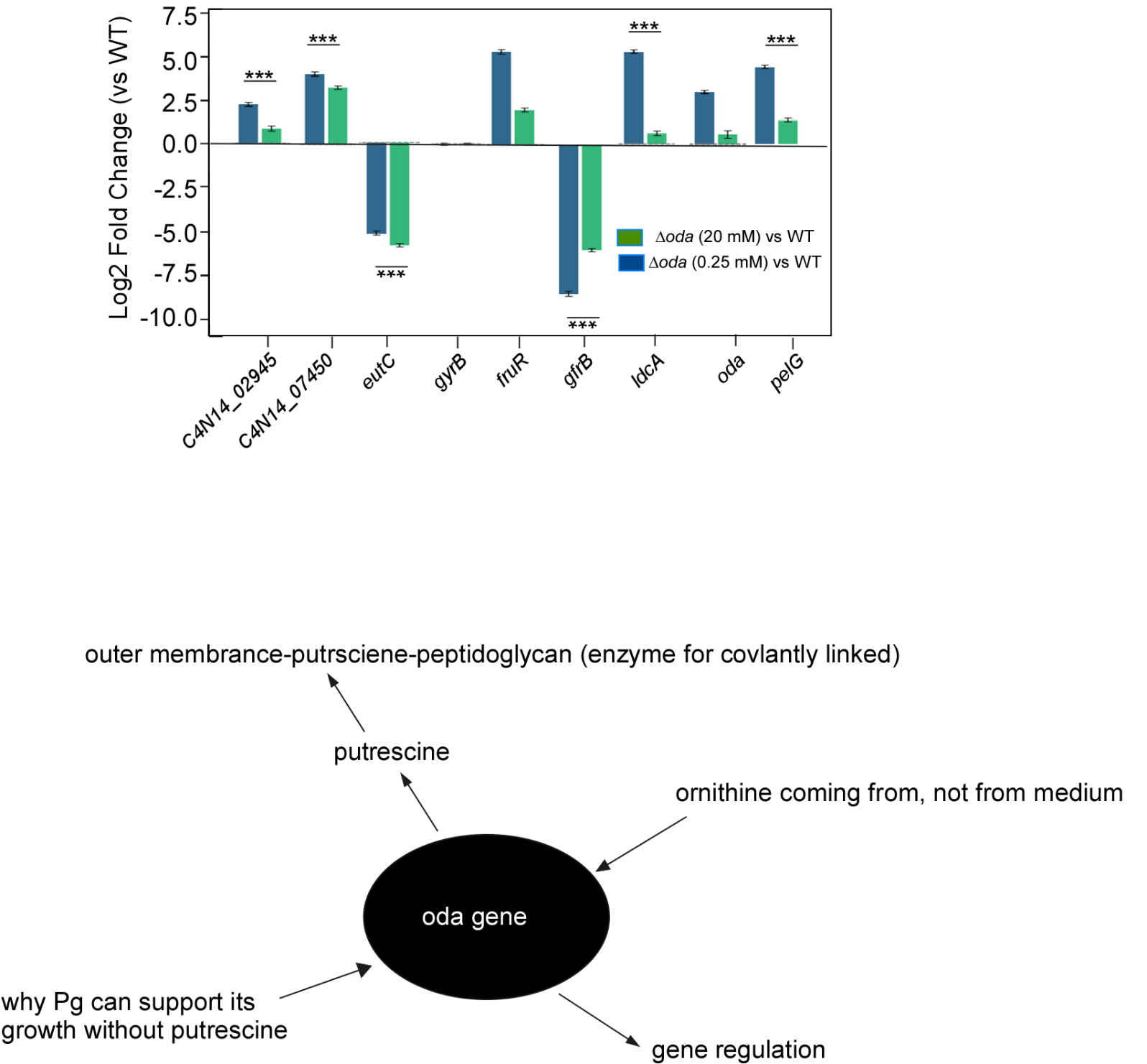


**Figure S2. qRT-PCR validation of selected genes.**
Relative expression of several genes, including *oda*, was compared between WT and Δ*oda* cells grown with 20 mM putrescine, and between Δ*oda* cells cultured with 0.25 mM versus 20 mM putrescine. The results are shown as means ± standard deviation (n=3), *<0.05, **<0.01, ***<0.0001 (student’s t-test).

**qRT-PCR**

WT and Δ*oda* strains were cultured in TSPC medium supplemented with either 20 mM or 0.25 mM putrescine, starting at an OD₆₀₀ of 0.1. After 10 h of growth, cells were harvested by centrifugation, resuspended in 1 mL TRIzol (Ambion), and lysed by mechanical disruption with 0.1-mm silica beads (MP Bio). Total RNA was extracted using the Direct-zol RNA MiniPrep kit (Zymo Research), and cDNA was synthesized with SuperScript III reverse transcriptase (Invitrogen). Quantitative PCR was performed with iTaq SYBR Green Supermix (Bio-Rad) using primers targeting C4N14_02945, C4N14_07450 (*pap2*), *eutC*, *oda*, *fruR*, *gfrB*, *ldcA*, and *pelG* (Table S2). Gene expression was calculated by the 2^–ΔΔCt method, normalized to *gyrB*. All assays were conducted in triplicate and validated in two independent experiments.

**Reference:**

1. Gc B, Zhou P, Wu C. 2023. HicA Toxin-Based Counterselection Marker for Allelic Exchange Mutations in *Fusobacterium nucleatum*. Applied and Environmental Microbiology:e00091-23.

2. Liu L, He Y, Zhang T, Geng R, Hu Y, Luo M, Zhou H, Liu X. 2024. Equip *Fusobacterium nucleatum* genetic tool kits with compatible shuttle vectors and engineered intermediatory E. coli strains for enhanced transformation efficiency. bioRxiv:2024.07. 17.603877.

3. Wu C, Al Mamun AAM, Luong TT, Hu B, Gu J, Lee JH, D’Amore M, Das A, Ton-That H. 2018. Forward genetic dissection of biofilm development by *Fusobacterium nucleatum*: novel functions of cell division proteins FtsX and EnvC. MBio 9:10.1128/mbio. 00360-18.

4. Nariya H, Miyata S, Suzuki M, Tamai E, Okabe A. 2011. Development and application of a method for counterselectable in-frame deletion in Clostridium perfringens. Applied and environmental microbiology 77:1375-1382.

5. Jones KR, Belvin BR, Macrina FL, Lewis JP. 2020. Sequence and characterization of shuttle vectors for molecular cloning in *Porphyromonas, Bacteroides* and related bacteria. Molecular oral microbiology 35:181-191.

6. Zhou P, GC B, Stolte F, Wu C. 2024. Use of CRISPR interference for efficient and rapid gene inactivation in *Fusobacterium nucleatum*. Applied and Environmental Microbiology 90:e01665-23.

7. Bibek GC, Wu C. 2025. The CarSR two-component system directly controls radD expression as a global regulator that senses bacterial coaggregation in *Fusobacterium nucleatum*. Journal of Bacteriology 207:e00529-24.
